# Evaluation of Barriers Contributing in the Demonstration of an Effective Nurse-Patient Communication in Educational Hospitals of Jahrom, 2014

**DOI:** 10.5539/gjhs.v6n6p54

**Published:** 2014-06-30

**Authors:** Marzieh Kargar Jahromi, Somayeh Ramezanli

**Affiliations:** 1 Community Health Nursing, Jahrom University of Medical Science, Jahrom, Iran; 2 Medical-Surgical Nursing, Jahrom University of Medical Science, Jahrom, Iran

**Keywords:** effective communication, nurse, patient, barriers

## Abstract

**Introduction:**

Establishing an effective communication with patients is an essential aspect of nursing care. Nurse-patient communication has a key role in improving nursing care and increasing patient’s satisfaction of health care system. The study aimed at evaluation of barriers contributing in the demonstration of an effective nurse-patient communication from their viewpoint.

**Method:**

This was cross-sectional study, carried out in 2014, with a sample of 200 nurses and patients drawn from two educational hospitals in jahrom city. Data were collected by using two questionnaire structured by the researchers. Data were analyzed using SPSS software (version 16).

**Result:**

The results of this study showed that the greatest barriers of nurse-patient communication were characteristics of nursing job with an average score of 71.05 ± 10.18.

**The most communication barriers from patients viewpoint including:**

heavy work load of the nurses, age, sex and language difference between patient and nurse and the spicy morality of nurses.

**Conclusion:**

It is concluded that overcome barriers to communication and support are needed to enable nurses to communicate therapeutically with patients in order to achieve care that is effective and responsive to their needs.

## 1. Introduction

Establishing an effective communication with patients is an essential aspect of nursing care. It is lamentable that some of the health care team members do not know how to make an effective relationship with the patients and do not devote any time for this necessity (Mohr, 2002).

The roles of patients and nurses in influencing communication processes are equally important (Tay, Hegney, & Ang, 2011). One important objective frequently stressed in patient’s care is to improve their satisfaction (Bengoa, Kawar, Key, Leatherman, & Saturno, 2006) and this is an important aspect of quality in any healthcare intervention (Oakland, 2004; Sofaer & Firminger, 2005). Researches on nursing care often complain about poor relationship (McCabe, 2004; Fakhr-Movahedi, Salsali, Negharandeh, & Rahnavard, 2011) and patients often feel health care personnel do not meet all their communication needs. According to the Research Center for Quality Care, 10.8% of patients believe that nurses sometimes or never listened to them carefully, do not explain things clearly and do not spend enough time with them (Agency for Healthcare Research and Quality, 2005). Because of asking questions or expressing their concerns, some patients have to face with inappropriate behavioral reactions of caregivers. Gaps in communication between caregivers and patients result in decreased quality of care, poor outcomes, and dissatisfaction with health care system (Bonds et al., 2003).

According to Merkouris’s study, patient satisfaction measurement can also be seen as a therapeutic intervention, an important criterion for making and evaluating organizational and administrative decisions (Merkouris, Ifantopoulos, Lanara, & Lemonidou, 1999). Patient satisfaction with nursing services gains even more importance, owing to the nature of nursing, patients may judge the overall quality of hospital services on the basis of their perceptions of the nursing care received (Yellen, Davis, & Ricard, 2002). Establishing a clear communication and information is obviously a prerequisite for the patient’s perception of satisfaction with the nursing care. On the other hand, there is much agreement on the importance of effective communication in patient care (Johansson, Olieni, & Fridlund, 2002).

Nurse- patient relationships are ideally towards decreasing stress in patient and her/his family and making them capable of self-care (Orem, 2001). Lack of communication and ineffective relationship often create problems in health care system, threatens professional credibility, and impose extra expenses on the patient and health care system (Potter & Perry, 2002). Effective communication between nurse and patient is an important factor into patient’s satisfaction, successful treatment, and patient’s acceptance of health care and treatment process which in turn increase the credibility of nursing profession in the society as well as nurses’ job satisfaction (Park & Song, 2005). The importance of good communication in the delivery of effective and appropriate nursing care has been well demonstrated by research and is reflected in policy documents (National Network for Cancer Research, 2012).

To improve health care and reduce patients’ dissatisfaction, the factors that affect communication between nurses and patients must be identified and taken into account; also response to all communication needs of patients should be provided (Rodin et al., 2009). Therefore, this study was conducted with the aim of evaluation of barriers contributing in the demonstration of an effective nurse-patient communication from their viewpoint.

## 2. Material and Method

A descriptive cross-sectional study was carried out at two university hospitals in jahrom (of Shiraz) from Jan to Feb 2014. The study population included the 100 patients hospitalized in the internal surgical wards and 100 nurses working in these wards of the two hospitals. The persons selected by proportional stratified random sampling technique. In the first step a total of 2 teaching hospitals that have the internal surgical wards selected as strata. Afterward sample size in each teaching-hospital was determined according to the proportion of the beds and nurses in wards in each hospital. We used the table of random numbers for selecting required sample from each hospital.

Data were collected by interviewing the patients and nurses based on two questionnaire structured by the researchers. 1-Questionnaire included five sections; first section, included demographic characteristics of the nurses and other four sections including questions on communication barriers–job characteristics (9 items), Individual and social factors (8 items), clinical condition of patient (4 items), and environmental factor (9 items). 2-Questionnaire included 2 sections; first section, included demographic characteristics of the patients and other section including 15 questions on communication barriers of nurse-patient. Standardized 5-point Likert scales ranging from strongly disagree to strongly agree (1 to 5 points) were used for all the items of questionnaires. Subsequently at every domain scores added together and according to the different number of questions in each domain, in order to compare scores with each other, scores were calculated based on 100. Higher score indicates a greater importance of communication barriers.

To assess the content validity, the questionnaires were sent to lecturers of the Nursing and Midwifery School and were edited according to their comments. To assess the reliability with test-retest method, 25 nurses and 25 patients filled out the questionnaires with one week interval. The correlation between the two measurements in nurses and patients questionnaire was accordingly 0.85 and 0.87 which were acceptable.

An ethical approval was granted from Jahrom University of Medical Sciences as well as from the directors and head nurses of the hospitals in Jahrom. Participants were informed about the purpose of the study and informed consent was obtained from all of them. Participation was entirely voluntary and the decision to participate or not to had no impact on the care of the patient.

Data were analyzed using SPSS software (version 16), descriptive statistical methods (frequency distribution, percentage, and mean) and correlation coefficient test.

## 3. Result

The mean age of nurses was 29/36 ± 5/62. All of the nurses participating in the study had a bachelor degree, and their work experiences in wards ranged from a year and half to 9 years. 68% of them were female and 72% were married. The mean age and the hospitalization days of patient were 40/62± 4/25 and 3/2±1/06 respectively. Most patients were female (75%) and married (64%). According to Table 1, the most communication barriers from patients viewpoint including: heavy work load of the nurses, age, sex and language difference between patient and nurse and the spicy morality of nurses.According to this table and in relation with the responses of the nurses and patients to criteria as barriers, the spearman correlation coefficient test showed high degree of correlation between nurses and patients viewpoints about the barriers to establish effective communication (Table 1). In relation to criteria that assessed only by the nurses’ viewpoint, the mean score of job characteristics has a high priority than other barriers (Table 2). The result in Table 3 is shown that there were the relationship between job characteristic and environmental factors with age of nurses. The mean score of communication barriers significantly associated with age and work hours of nurses.

**Table 1:** The absolute frequency distribution of nurses and patients’ responses to common criteria as barriers to effective nurse-patient communication

Barriers to effective nurse-patient communication		Nurses	Patients
1	Age difference between patient and nurse	**41**	**48**
2	Gender difference between patient and nurse	**26**	**34**
3	language difference between patient and nurse	**57**	**69**
4	The class differences between patients and nurse	**7**	**12**
5	Outside problems in the work environment of nurse	**25**	**20**
6	Unfamiliarity of patients to nurses duties	**24**	**20**
7	The spicy morality of nurse	**27**	**45**
8	Exceeded expectations of patient from nurse	**21**	**15**
9	heavy work load of the nurses	**75**	**58**
10	Contact of patients with different mentality of nurses	**20**	**16**
11	History of previous hospitalization	**12**	**8**
12	Being visitor of patient	**23**	**21**
13	Transmissible diseases of patient	**32**	**30**
14	Lack of facilities (welfare - therapeutic) for patients	**31**	**25**
15	Unsanitary room of patient correlation coefficient	**15**	**12**
		r=0/94	P<0001

**Table 2:** The mean score of nurses’ opinions on barriers to effective nurse - patient communication

Barriers to effective nurse-patient communication	M	SD
Individual and social factors	71.05	10.18
Job Characteristics	78.24	9.80
Clinical condition of the patient	69.15	13.81
Environmental factors	71.68	12.38
Total	72.53	7.97

**Table 3:** The correlation between mean score of nurses’ opinions on barriers to effective nurse - patient communication with their age and work time

Barriers to effective nurse-patient communication	Age correlation coefficient	work time correlation coefficient
Individual and social factors	r=- 0.10	r=- 0.05
Job Characteristics	r=0.30**	r=- 0.10
Clinical condition of the patient	r=- 0.07	r=- 0.10
Environmental factors	r=- 0.40**	r=- 0.20*
Total	r=- 0.30**	r=- 0.20**

Note: significance level of Spearman correlation test for numbers that are marked with

* and

** considered by P<01 and P<05 respectively.

## 4. Discussion

Effective communication between nurses and patients is one of the most important elements for improving patient satisfaction, treatment compliance and health outcomes (Shukla, Yadav, & Kastury, 2010). While in the most of developed countries defined standards are applied in nurse - patient communication. However, obvious standards consistent with the conditions in Iran are not defined. The studies conducted about the relation of nurse and patient in Iran showed problem in this regard. For example, Abedi et al., found that the communication of the nurse and patient is bad and by presenting educational plans for the patients, it can be improved (Abedi, Alavi, Aseman Rafat, & Yazdani, 2005).

The results of this study showed the greatest barrier of nurse - patient communication was characteristics of nursing job and there was a significant correlation between these barriers with age of the nurses. It was found that “heavy work load of the nurses”; being busy with the ward’s routine works, “physical and mental fatigue” and “deficiency of welfare facilities for nurses” are main barriers of effective communication that nurses have more emphasis on it. Lahuti in his research has found that the deficiency of nurse than patient and lack of adequate time for patients were the most important barriers of communication with patients that implies on it. The research also mentioned fatigue-related nurse’s work is other barrier in communication with patients (Lahoti, 1996).

Nurses must learn to manage time effectively in order to complete the many and varied tasks that fall under their responsibility. By spending time with the patient, the nurse allows the patient to feel cared for, valued, and ideally, understood. When the busy nurse is unable to spend time with the patient, that patient may feel that he is not important and his needs may not be met. Finding ample time to avoid appearing rushed, to gather important diagnostic facts, to educate patient, and to establish therapeutic relationships remains a significant challenge for every nurse (DeLaune & Ladner, 2002). Stress and pressure from time constraints often cause miscommunication, flawed assumptions, decreased nurse and patient satisfaction, and poor or nonexistent care coordination (Chapman, 2009). We can say that the factors mentioned above, not permit to effective communication of them with patients. Meanwhile their work is hard and boring; often not perform a proper appreciation of nurses. In this situation can be expected that it’s not a desirable quality of nurse-patient communication and the combination of these factors will affect nursing morality. The patient reported spicy morality of nurses as a barrier to communication. Thus we describe nursing morality as existing in real human social space, in a specific historical time and context. We also note that in order to understand why nurses’ morality became problematic, we must first gain an insight into the sociocultural world in which their morality was constructed. Historically, nurses were obliged to act according to the policies and directives issued by authorities in all fields of health care (Walker, 1998).

The second group of barriers from the nurse’s point of view was related to nurses’ working conditions and environment.These obstacles can be categorized in two groups: obstacles related to environment such as crowded wards and rooms, and obstacles related to the working conditions such as having very sick patients in the ward. The main obstacle related to the nurses’ working conditions and environment was that of crowded wards and rooms. In the educational hospitals, the high number of medical and nursing students in the wards can turn to an obstacle in nurse-patient relationship (Bakhtiari, Mohammadzadeh, & Moshtaq, 2009). In this area, nurses considered that lack of participation in workplace decisions, not appreciating of managers of them and feeling of injustice in the workplace, were the most important barriers to communication. Meehan et al found similar results in their study (Meehan et al., 2011).

The environmental factor of communication for nurses includes multidisciplinary team practice and inter-professional working, across different care settings, within a safe environment. In addition, it has been suggested that improving communication requires a detailed understanding of the setting and context in which patient care is delivered and a commitment on behalf of a healthcare organization to a quality improvement, such as supporting team-based delivery of care (Oandasan et al., 2006). In another study, the presence of a supporting environment, revision in manager’s rules and adequate resources to improve the relation between the nurse and patient is emphasized (Clancy, 2009). Thus, the hospital administers should highly take into consideration the working environment and conditions of the clinical nurses.

In the present study, nurses identified having very sick patients in the ward as communication barrier. Some researchers have been reported the deterioration and poor prognosis of the disease, limit the nurse-patient communication. In this regard, Kruijver and colleagues in their study conclude that the associations of nurses in cancer patients are limited (Kruijver, Kerkstra, Bensing, & van deWiel, 2000).

According to result of our study, the most communication barriers from patient’s viewpoint including: heavy work load of the nurses, age, sex and language difference between patient and nurse and the spicy morality of nurses.

Gender difference between patients and nurses is an indicator that Patients mentioned as an influential obstacle for communication. This indicates that nurses are less affected than patients by gender in job performance. However cultural and religious status of the region of the study justify patients’ point of view, influence of gender in nurse-patient relationship may differ according to culture and beliefs of each community. Although gender differences mentioned as a barrier for communication with nurses in the Meehan study, it was difficult for both male and female patients to communicate with male nurses (Meehan et al., 2011).

Another factor that patients emphasize more than nurses was language difference between patient and nurse. Language difference affects the quality of nurse-patient communication (Ferguson & Candib, 2002). Because nurses are responsible for initiating and continuing relationship with patients, so it can be claimed that they do their professional duties such as communication with patients regardless of their social class and age (Registered Nurses Association of British Columbia, 2003).

Thus, improvements towards effective communication in healthcare settings involve synchronizing efforts across the two levels that is, the individual, and the organization.

1) The individual Human factors, such as fatigue and stress levels of staff, personality and attitudes, distractions and interruptions have been reported to influence the effectiveness of communication (Zeltser & Nash, 2009).
2) Leadership support—Communication within the health service can be improved with an organization providing strong leadership, through implementation of policies and procedures and identifying clinical leaders to drive improvements in welfare facilities for nurses (Leonard, Graham, & Bonacum, 2004).

## 5. Conclusion

Effective communication is the main feature of nursing care and it is considered as a necessity over the time. To communicate effectively, we need to be recognizing the barriers. Nurses must communicate effectively in order to perform their roles as educator, case manager, and active member of the health care team. When we are attentive of them, these factors will help us plan, analyze conditions, solve problems, and in general do better in our work. It is concluded that overcome barriers to communication and support are needed to enable nurses to communicate therapeutically with patients in order to achieve care that is effective and responsive to their needs.

## Study Limitation

The limitation of the study is the lack of generalization of the findings to other studies like other quality studies.
